# Ultrasonographic Tongue Base Motion Does Not Correlate With Hypoglossal Nerve Stimulation Outcomes

**DOI:** 10.1002/lio2.70376

**Published:** 2026-03-10

**Authors:** Samuel Tschopp, Vlado Janjic, Marco Caversaccio, Urs Borner, Kurt Tschopp

**Affiliations:** ^1^ Department of Otorhinolaryngology, Head and Neck Surgery Kantonsspital Baselland Liestal Switzerland; ^2^ Department of Otorhinolaryngology, Head and Neck Surgery, Inselspital, University Hospital and University of Bern Bern Switzerland

**Keywords:** hypoglossal nerve stimulation, obstructive sleep apnea, treatment outcome, ultrasonography, upper airway stimulation

## Abstract

**Objective:**

To evaluate whether tongue base motion patterns, assessed by submental ultrasonography during unilateral hypoglossal nerve stimulation (HNS), are associated with treatment response in obstructive sleep apnea patients.

**Methods:**

This cross‐sectional study included 64 patients with unilateral HNS. Standardized submental B‐mode ultrasound was performed to assess tongue‐base motion under awake stimulation. Two independent, blinded raters evaluated the magnitude of anterior tongue base movement in the axial and sagittal planes. Further, the symmetry of bilateral tongue protrusion and tongue shape was classified. The primary endpoint was the correlation between apnea‐hypopnea (AHI) reduction and ultrasonographic findings.

**Results:**

Ultrasonographic assessment showed substantial to excellent agreement between the two raters. No significant correlation was found between AHI reduction and anterior tongue displacement in either the axial (Spearman's *ρ* = 0.20, *p* = 0.12) or sagittal plane (*ρ* = −0.08, *p* = 0.64). Clinically meaningful bilateral activation was present in 33% of patients but was not associated with treatment outcome (*ρ* = 0.03, *p* = 0.84). Neither tongue shape was predictive of AHI improvement (buckling *p* = 0.13 and trough sign *p* = 0.79). Tongue movement patterns were not associated with stimulation voltage.

**Conclusion:**

Ultrasonographic assessment of tongue base motion under HNS does not correlate with treatment response. Neither symmetry, magnitude, nor qualitative shape was associated with AHI reduction. These findings suggest that visible tongue motion may not reliably reflect functional improvement in the airway. Future research should define the role of ultrasound in HNS evaluation and optimization.

**Level of Evidence:**

3b.

**Trial Registration:**

ClinicalTrials.gov Identifier: NCT06154577

## Graphical abstract

Ultrasonographic tongue‐base motion during hypoglossal nerve stimulation showed no meaningful association with treatment response in obstructive sleep apnea. Neither displacement magnitude, symmetry, nor qualitative tongue shape correlated with AHI reduction, despite good interrater agreement. Ultrasound‐visible tongue motion therefore appears to have limited value for predicting or assessing HNS efficacy.

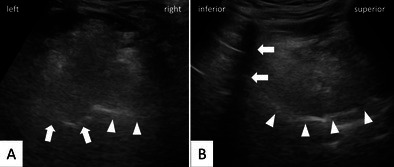

## 1 Introduction

Obstructive sleep apnea (OSA) is a prevalent sleep‐related breathing disorder characterized by recurrent upper airway collapse during sleep, resulting in intermittent hypoxemia, increased cardiovascular risk, and excessive daytime sleepiness [1, 2, 3, 4, 5]. Hypoglossal nerve stimulation (HNS) has emerged as an alternative treatment for patients with moderate‐to‐severe OSA who are unable to tolerate continuous positive airway pressure (CPAP) therapy [6, 7, 8, 9, 10]. By activating the tongue protrusor muscles, predominantly the genioglossus muscle, HNS enlarges the posterior airway space at the base of the tongue. Clinically, differences in tongue motion under stimulation are observed. Variations in hypoglossal nerve anatomy, electrode placement, and stimulation parameters can influence the recruitment of specific muscle groups, including tongue retractors, resulting in distinct patterns of tongue movement. Additionally, the predominance of particular nerve fibers and muscle orientations may result in distinct tongue shapes during stimulation. Despite relatively strict selection criteria for implantation, suboptimal treatment responses remain frequent [8]. Evaluating tongue muscle activation patterns could yield insights into upper airway mechanics under stimulation.

During implantation and titration, tongue movement is typically observed through the mouth. However, only the anterior tongue is visible, while the tongue base, which is clinically more relevant for airway patency, remains obscured to routine examination. Using wake and drug‐induced sleep endoscopy, Heiser et al. [11] demonstrated that bilateral tongue protrusion leads to improved retropalatal opening through palatopharyngeal coupling. This has led to the hypothesis that bilateral tongue protrusion might improve HNS outcomes, as residual retropalatal obstruction is common among non‐responders [8]. While bilateral HNS systems aim to achieve symmetrical activation, contralateral muscle engagement via cross‐innervation of the genioglossus muscle is observed in approximately 39% of patients with unilateral HNS [12]. Electrophysiological measurements by Steffen et al. [13] also demonstrated bilateral genioglossus activation with unilateral stimulation. Thus, unilateral HNS may still produce bilateral tongue motion. Currently, it remains unclear whether true bilateral stimulation offers clinical advantages over unilateral activation [13, 14].

Importantly, visible anterior tongue protrusion through the mouth may not accurately reflect posterior airway opening, since tongue protrusion may not always dilate the airway [15]. Using submental ultrasonography, Fleury Curado et al. [15] reported that a preserved tongue shape during protrusion was associated with better response to multichannel targeted HNS. Other studies have shown that backscattered ultrasound imaging for tissue characterization can predict OSA severity [16] and response to HNS [17]. B‐mode ultrasound is a noninvasive, real‐time modality for dynamically evaluating tongue base motion. Ultrasound may serve as a valuable tool for assessing stimulation‐induced upper airway dynamics during implantation and titration.

This study investigates whether the extent and pattern of tongue base motion, as visualized with submental B‐mode ultrasound during awake HNS, correlate with treatment response. The authors hypothesize that tongue movement patterns are associated with therapeutic outcomes.

## 2 Materials and Methods

The study received approval from the local ethics committees (Ethikkommission Nordwest‐und Zentralschweiz EKNZ und Ethikkommission Bern, project ID 2023‐00117) and was conducted in accordance with the Declaration of Helsinki [18]. The study is registered on ClinicalTrials.gov.

For this study, we assumed that HNS settings, electrode placement, and baseline patient characteristics influence tongue base motion, thereby affecting treatment outcomes. Tongue motion was not considered the sole determinant of outcome, as baseline characteristics may also exert direct effects that are not reflected in observable differences in tongue motion. These assumed causal relationships are illustrated in the directed acyclic graph shown in Figure S1.

### 2.1 Study Design and Participants

This cross‐sectional study consecutively recruited patients with unilateral HNS undergoing routine clinical follow‐up at two centers: a tertiary care hospital (Inselspital, Bern University Hospital and University of Bern) and a secondary care hospital (Kantonsspital Baselland, Liestal). All patients who underwent unilateral HNS implantation (Inspire II, Inspire Medical Systems, MN, USA) were eligible for inclusion. Patients were excluded if they had a history of neck surgery other than HNS implantation or tonsillectomy due to potential postoperative anatomical alterations. Additional exclusion criteria included congestive heart failure, chronic obstructive pulmonary disease, comorbid sleep disorders, and pregnancy. All participants provided written informed consent.

### 2.2 Follow‐Up Examination and Sleep Testing

All participants underwent routine clinical follow‐up. Pre‐ and postoperative home sleep apnea testing were performed using either respiratory polygraphy (Nox T3, Nox Medical, Reykjavik, Iceland) or peripheral arterial tonometry (WatchPAT, Itamar Medical, Caesarea, Israel). Postoperative sleep studies were conducted under optimally titrated therapeutic stimulation. If stimulation parameters were adjusted during the follow‐up visit, sleep studies were repeated to ensure identical stimulation parameters for ultrasonographic evaluation and sleep testing.

### 2.3 Ultrasonographic Assessment

Ultrasound evaluation was performed during wakefulness in a supine position using a portable ultrasound system (Terason uSmart3200T, Teratech Corporation; FDA approval K193510) with a 2–5 MHz convex transducer (5C2A). To standardize image acquisition, the transducer was aligned to the patient's head and neck using a laser‐guided alignment system (AmCad BioMed Corporation; FDA Establishment Registration & Device Listing No. 3015218501, Figure S2). Stimulation parameters were set to the patient's therapeutic settings. Each patient underwent three ultrasound scans in both the axial and sagittal planes over separate stimulation cycles.

Two independent, blinded raters (K.T. and V.J.) assessed all recordings using a standardized worksheet (Supporting Information Methods). Each rater assigned a single summary score to each parameter for each patient. In the axial plane, the anterior tongue base movement of the implanted side was rated on a visual analog scale (VAS) from 1 (*none*) to 5 (*very good*). In the sagittal plane, the anterior movement of the tongue base was rated analogously. Bilateral tongue base movement was rated in the axial plane with reference to the implanted side on a visual analog scale (VAS) from 1 (*isolated unilateral*) to 5 (*completely bilateral*). Clinically meaningful bilateral activation of the tongue base was defined as a VAS score ≥ 3. The engagement of the transverse and vertical intrinsic tongue muscles results in a midline V‐shaped contraction of the tongue base, denoted as the “trough sign”. This was noted as either present or absent [19]. Additionally, the sagittal motion pattern was evaluated for the presence or absence of “buckling,” defined as tongue elevation rather than forward movement, as described by Fleury Curado et al. [15].

### 2.4 Statistical Analysis

All statistical analyses were performed in R version 4.5.0 (R Foundation for Statistical Computing, Vienna, Austria). Descriptive statistics were calculated for the baseline and follow‐up patient characteristics. Categorical variables are summarized as counts and percentages. Interrater reliability for ultrasonographic assessments was assessed using the intraclass coefficient for continuous variables and Cohen's kappa for binary data. Agreement was interpreted according to Landis and Koch as poor (< 0.20), fair (0.21–0.40), moderate (0.41–0.60), substantial (0.61–0.80), and excellent (> 0.81) [20]. The primary endpoint was the absolute reduction in apnea‐hypopnea index (AHI) from preoperative to postoperative assessment. Associations between AHI reduction and continuous ultrasonographic parameters were evaluated using Spearman's rank correlation coefficient with corresponding 95% confidence intervals [21]. Binary features, such as the presence or absence of buckling and trough sign, were compared using the Mann–Whitney *U* test, with effect size reported as the Hodges‐Lehmann median difference and 95% confidence interval. To avoid confounding from electrode configuration, analyses involving stimulation parameters were restricted to patients with the standard configuration (+/−/+) [22].

To assess potential confounding due to positional changes, as ultrasound was acquired during wakefulness in the supine position, we performed a sensitivity analysis using positional AHI. We repeated the primary association analyses for reduction in supine AHI and non‐supine AHI as outcomes. A second sensitivity analysis was performed by stratifying according to responder status. To account for multiple testing, the Benjamini–Hochberg procedure was applied. Data was visualized using scatter plots with locally estimated scatterplot smoothing (LOESS) and 95% confidence intervals to illustrate trends.

## 3 Results

### 3.1 Patient Cohort

Sixty‐five consecutive patients were recruited. One patient was excluded due to insufficient image quality, resulting in 64 patients included in the final analysis. The median time from implantation to examination was 21.5 months (interquartile range [IQR]: 5.4 to 43.4 months, minimum 2 months, maximum 8.9 years). Table 1 summarizes pre‐ and postoperative patient characteristics, including sleep study results, Epworth Sleepiness Scale scores [23], and snoring intensity. According to the Sher criteria, 42% (*n* = 27/64) of patients were classified as responders [24]. The median therapeutic stimulation amplitude was 1.7 V (IQR: 1.2–2.2 V). The standard electrode configuration (+/−/+) was used in 86% (*n* = 55) of patients, while 5% (*n* = 3) of patients used a −/0/− monopolar configuration, 5% (*n* = 3) a 0/−/0 monopolar configuration, and 2% (*n* = 1) patient used −/+/−.

**TABLE 1:** Basic patient demographics.

Variable	Preoperative	Postoperative	Median difference	*p*
Body mass index (kg/m2)	27.6 [26.3, 30.4]	28.4 [26.6, 29.9]	0.0 [−0.8, 0.5]	0.80
Apnea‐hypopnea index (events per hour)	40.5 [30.9, 57.2]	21.0 [11.1, 35.7]	18.5 [14.4, 22.5]	0.00
Supine apnea‐hypopnea index (events per hour)	47.6 [29.7, 67.9]	26.7 [19.4, 48.0]	15.4 [8.5, 23.0]	0.00
Non‐supine apnea‐hypopnea index (events per hour)	47.9 [31.5, 70.5]	14.8 [7.2, 26.6]	34.3 [26.4, 43.3]	0.00
Oxygen desaturation index (events per hour)	23.0 [15.5, 34.2]	12.2 [6.7, 24.1]	10.1 [6.3, 14.1]	0.00
Respiratory disturbance index (events per hour)	29.4 [19.5, 44.2]	26.3 [17.3, 42.0]	4.9 [1.7, 8.3]	0.00
Mean oxygen saturation (%)	92.2 [91.9, 92.8]	91.0 [89.5, 92.7]	1.3 [−0.6, 2.8]	0.11
Time below 90% oxygen saturation (% of sleep time)	3.2 [1.0, 9.8]	1.1 [0.1, 4.5]	3.7 [1.3, 7.4]	0.01
Epworth Sleepiness Scale	10.5 [7.0, 13.0]	6.0 [4.0, 9.8]	3.5 [2.5, 5.0]	0.00
Snoring, VAS	8.0 [6.0, 9.0]	3.0 [1.0, 6.0]	3.5 [3.0, 4.5]	0.00

*Note:* The median and interquartile range, along with the Mann–Whitney *U* test for significance, are reported. VAS, visual analog scale from 0 to 10.

**FIGURE 1:**
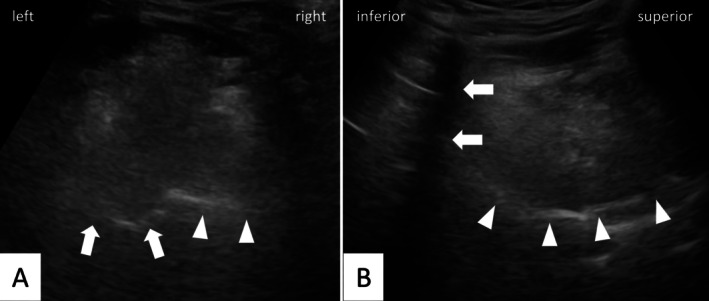
Ultrasonographic still image from the recorded sequences in the axial (A) and sagittal (B) planes of the tongue base. In the axial plane (A), the right implanted tongue base to airway interface is indicated with arrowheads, and the left non‐implanted side is indicated by arrows. On the sagittal view (B), the tongue‐airway interface is indicated with arrowheads and the hyoid shadow using arrows.

Tongue movement assessments were conducted by two independent raters, blinded to patient data, using video recordings. An exemplary still image is provided in Figure 1, and movement patterns are illustrated in the Video S1. Table 2 presents the detailed scoring results and interrater reliability metrics.

**TABLE 2:** Overview of movement patterns and agreement.

Variable	Rater 1, mean (SD)	Rater 2, mean (SD)	Rater 1, median (IQR)	Rater 2, median (IQR)	Agreement (95% CI)
Axial tongue base movement (VAS)	3.5 (1.1)	3.3 (1.0)	3.5 [3.0, 4.0]	3.0 [3.0, 4.0]	0.79 (0.68, 0.87)
Sagittal tongue base movement (VAS)	3.3 (1.2)	3.4 (1.0)	3.0 [2.0, 4.0]	3.0 [3.0, 4.0]	0.75 (0.57, 0.86)
Bilateral movement (VAS)	2.3 (1.5)	2.2 (1.0)	2.0 [1.0, 4.0]	2.0 [1.0, 3.0]	0.60 (0.41, 0.73)
Trough sing (yes/no)	—	—	—	—	0.85 (0.70, 0.99)
Buckling (yes/no)	—	—	—	—	0.92 (0.77, 1.00)

*Note:* Intraclass coefficient was used for continuous data, and Cohen's kappa for binary data. VAS, visual analog scale from 1 to 5.

### 3.2 Anterior Tongue Base Movement

**FIGURE 2:**
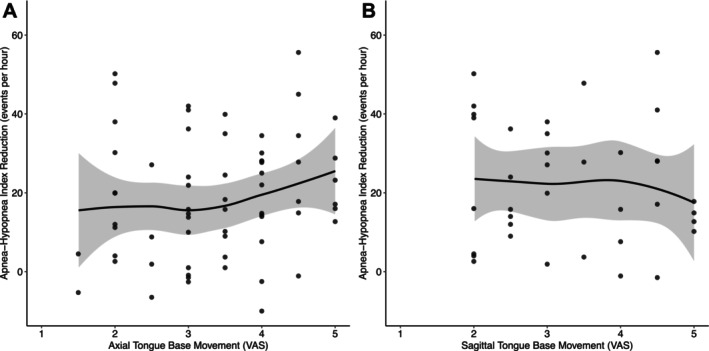
Anterior tongue base movement. On an axial (A) plane, the tongue base movement in an anterior direction on the implanted side is graded on a visual analog scale (VAS, A) from 1 (*no movement*) to 5 (*maximum movement*). On the sagittal (B) scan, the anterior movement of the tongue base is rated analogously. A locally estimated scatterplot smoothing (LOESS) with a 95% confidence interval (gray shading) illustrates the trend.

No significant correlation was found between the reduction in AHI and the magnitude of anterior tongue base movement. In the axial plane, Spearman's *ρ* was 0.20 (95% CI: −0.06 to 0.46, *p* = 0.12, *p* adjusted = 0.33; Figure 2A). In the sagittal plane, the correlation was negligible (*ρ* = −0.08, 95% CI: −0.44 to 0.28, *p* = 0.64, *p* adjusted = 0.84; Figure 2B).

### 3.3 Bilateral Tongue Base Movement

**FIGURE 3:**
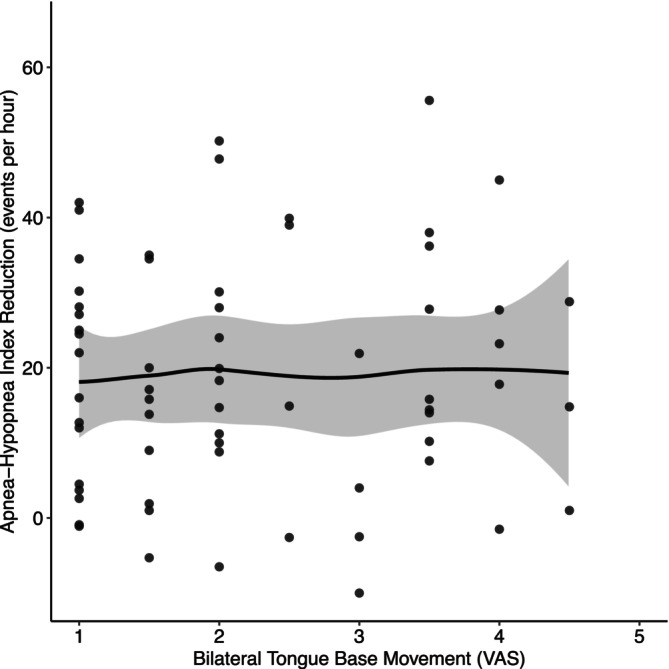
Bilateral tongue base movement. The pattern is graded on a visual analog scale (VAS) from 1 (*exclusively unilateral movement*) to 5 (*symmetrical bilateral movement*). A locally estimated scatterplot smoothing (LOESS) with a 95% confidence interval (gray shading) illustrates the trend.

Clinically meaningful bilateral tongue base activation, defined as a VAS score of 3 or greater, was observed in 33% (21/64) of patients. However, a significant association was found between the degree of bilateral activation (VAS) and AHI reduction (*ρ* = 0.03, 95% CI: −0.23 to 0.28, *p* = 0.84, *p* adjusted = 0.84; Figure 3).

### 3.4 Tongue Shape Assessment

**FIGURE 4:**
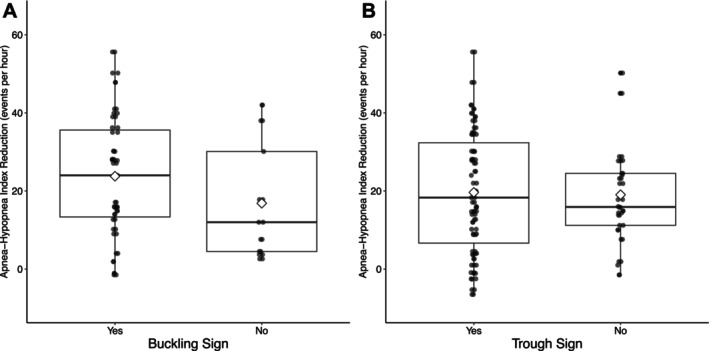
Buckling and trough sign. A buckling (A) of the tongue on a sagittal ultrasound scan or a trough sign (B) on an axial scan is graded as present or absent against the apnea‐hypopnea index reduction. A diamond shape indicates the mean.

Buckling of the tongue was observed in 22% (*n* = 8/36) and 25% (*n* = 9/36) of cases, as reported by the two raters, with excellent interrater agreement. The presence of buckling was not significantly associated with AHI reduction (median difference 8.2 events/h, 95% CI: −2.0 to 16.0, *p* = 0.13, *p* adjusted = 0.33; Figure 4A).

The trough sign, representing intrinsic tongue muscle activation, was observed in 30% (*n* = 19/63) and 30% (*n* = 19/64) of patients, with excellent interrater agreement. There was no significant association between the presence of the trough sign and AHI reduction (median difference 0.5 events/h, 95% CI: −5.6 to 6.7, *p* = 0.79, *p* adjusted = 0.84; Figure 4B).

### 3.5 Sensitivity Analyses

Results remained unchanged in all sensitivity analyses. When positional AHI reductions were analyzed separately, no significant associations were observed between non‐supine (Table S1a) and supine (Table S1b) AHI reduction and any ultrasonographic parameter. Restricting the analysis to responders likewise demonstrated no significant correlations between tongue base motion patterns and AHI reduction (Table S2).

### 3.6 Stimulation Parameters and Tongue Movements

For the analysis of stimulation parameters, only patients with the standard configuration (+/−/+) were included to avoid confounding from electrode configuration. Tongue base movements showed no association with the therapeutic amplitude. Anterior displacement in the axial (*ρ* = 0.14, 95% CI: −0.14 to 0.43, *p* = 0.29) and sagittal planes (*ρ* = 0.06, 95% CI: −0.28 to 0.41, *p* = 0.73; Figure S3) was independent of stimulation amplitude. Bilateral activation was likewise independent of stimulation voltage (*ρ* = −0.02, 95% CI: −0.29 to 0.28, *p* = 0.91). Voltage levels also did not significantly differ based on the presence of buckling (median difference 0.2 V, 95% CI: −0.1 to 0.5, *p* = 0.17) or trough sign (median difference: −0.1 V, 95% CI: −0.3 to 0.3, *p* = 0.68, Figure S4).

## 4 Discussion

In this study, ultrasonographic assessment of tongue base motion during HNS in awake patients showed no association with treatment response, as measured by AHI reduction. Neither the magnitude nor the configuration of tongue base displacement correlated with therapeutic efficacy. Additionally, bilateral tongue base protrusion did not yield better results than unilateral tongue motion. These results remained unchanged in the sensitivity analyses.

Contrary to our initial hypothesis, greater anterior displacement of the tongue base did not correlate with improved AHI reduction. Although prior studies have demonstrated the feasibility of using ultrasound to assess tongue movement under HNS [15, 25, 26], our findings suggest that visible displacement may not reflect functional airway improvement. Hofauer et al. [25] reported that a tongue protrusion of 1 cm or more results in a significant reduction in AHI. Fleury Curado et al. [15] used midsagittal ultrasound to assess tongue motion in HNS patients during wakefulness. They found tongue protrusion with preserved shape predicted improved airway patency in non‐REM sleep. Anterior displacement with increased height, described in our study as “tongue buckling”, was linked to reduced patency [15]. In our cohort, tongue buckling was not associated with any significant difference in AHI reduction.

We also identified the trough sign, a midline depression indicative of intrinsic tongue muscle activation. To our knowledge, this is the first study to systematically assess this ultrasonographic feature. Despite its distinct appearance and theoretical relevance, the trough sign was not associated with treatment response in our cohort.

Schwab et al. [27], using computed tomography in awake patients, found that responders to HNS had greater anterior tongue motion and retroglossal airway expansion. However, our findings suggest that improvements in airway patency may occur independently of visible tongue base motion. It is plausible that the tonicity of the upper airway musculature, rather than displacement, is sufficient to achieve a therapeutic effect [28, 29]. Preventing obstruction by stabilizing the airway may be sufficient, and anterior tongue movement may be unphysiological and unnecessary for improving outcomes. Another explanation for our findings is that, despite opening the retroglossal airway, an obstruction may persist at other levels, such as the palate or the lateral oropharyngeal walls, as supported by previous studies [30, 31, 32, 33].

The findings underscore the need for caution among sleep physicians when adjusting stimulation amplitudes, as higher settings intended to enhance airway mechanics may compromise patient comfort [34]. Pawlak et al. [35] propose adjustments to the electrode configuration and impulse strength under endoscopic visualization in patients with suboptimal results. Steffen et al. [22] demonstrated that stimulation amplitude can be reduced while maintaining therapeutic efficacy by adjusting pulse width and frequency. We fully agree and would caution against increasing stimulation levels beyond patients' comfort in the hope of achieving better therapeutic results. Furthermore, it is important to note that ultrasound can visualize only soft tissue and the airway interface but cannot directly measure the airway opening. Therefore, ultrasound and awake or drug‐induced endoscopy should be used in conjunction.

Bilateral tongue base activation remains of special interest. In our cohort, approximately one‐third of patients demonstrated clinically meaningful bilateral activation, consistent with the findings of Sturm et al. [12], who observed contralateral activation via cross‐innervation in 39% of cases using electromyography. Similar to our results, they reported no significant difference in clinical outcomes between unilateral and bilateral activation [12]. These data support the notion that unilateral HNS can achieve functional bilateral effects [13, 14].

### 4.1 Strengths

This study introduces a novel, standardized approach to submental B‐mode ultrasonography for evaluating tongue base movement during HNS. Ultrasound is widely accessible and quick to perform. To our knowledge, it is the first investigation to systematically assess the clinical relevance of unilateral versus bilateral tongue base activation using ultrasound. The use of a laser‐guided alignment ensured high reproducibility and consistency in image acquisition, enhancing the methodological rigor. The blinded design with two independent raters further strengthens the reliability and validity of the ultrasonographic assessments.

### 4.2 Limitations

Several limitations must be acknowledged. First, follow‐up assessments were conducted at variable time points after HNS implantation, reflecting routine clinical practice rather than predefined intervals. Second, due to the dynamic and complex nature of tongue motion, precise quantitative measurements were not feasible; instead, movement was graded using a visual analog scale by two independent raters. Third, variability in both sleep testing and ultrasonographic assessment may have affected the accuracy of outcome correlations [36]. Fourth, ultrasound visualizes the soft‐tissue‐airway interface but lacks information about the airway lumen. Conversely, awake endoscopy and DISE assess the airway lumen but not the surrounding soft tissue. Therefore, we consider ultrasound and endoscopy complementary modalities. Fifth, since only the therapeutic settings were tested, no inference can be made about configuration and impulse strength to ultrasound assessment and therapy response. Sixth, the relatively low responder rate limits the generalizability of our findings. Finally, the observational design and exploratory nature of the analysis preclude definitive causal inference. Although multiple comparisons were performed, the false discovery rate was controlled using the Benjamini–Hochberg procedure.

### 4.3 Future Research

Currently, our findings do not provide evidence for the routine clinical use of ultrasonography based solely on tongue movements. Future studies should focus on the utility of submental B‐mode ultrasound imaging during HNS implantation and follow‐up titration. Ultrasound is readily available and may provide rapid, valuable insights during postoperative titration and device optimization. Particular emphasis should be placed on characterizing dynamic changes in tongue morphology, characterizing response to different electrode configurations and impulse strengths, and identifying activation patterns of specific muscle groups. Ultrasound with shear‐wave elasticity imaging or backscattered imaging could further enhance our understanding by enabling noninvasive, real‐time assessment of muscle activation without the need for electromyography [16, 17, 19]. Additionally, awake endoscopy should be performed in conjunction with ultrasound to directly compare the information gained and corroborate findings of both modalities. Large, prospective studies are needed to validate these methods and clarify their potential to improve patient selection and therapeutic outcomes in HNS.

## 5 Conclusion

This study found no significant association between the magnitude, symmetry, or shape of tongue base motion and treatment response to unilateral HNS. These findings challenge the assumption that more pronounced or bilateral tongue motion improves therapeutic outcomes and support the notion that airway patency may be achieved through mechanisms independent of visible anterior tongue displacement. While submental B‐mode ultrasound allows non‐invasive, standardized visualization of tongue dynamics, its clinical utility for predicting HNS efficacy remains unproven. Furthermore, large prospective studies are needed to determine whether ultrasonographic parameters can improve patient selection and treatment optimization in HNS.

## Funding

AmCad Biomed Corporation, Taipei, Taiwan, funded the ultrasonography measurements for this study.

## Ethics Statement

All procedures performed in this study involving human participants were in accordance with the ethical standards of the Swiss Association of Research Ethics Committees (Ethikkommission Nordwest‐und Zentralschweiz EKNZ und Ethikkommission Bern, project ID 2023‐00117) and with the 1964 Helsinki Declaration and its later amendments or comparable ethical standards.

## Conflicts of Interest

Kurt Tschopp received grants from Amcad Biomed Corporation, Taipei, Taiwan, during this study.

## Supporting information


**Figure S1:** Directed acyclic graph illustrating the assumed causal relationships between baseline patient characteristics, hypoglossal nerve stimulation, and tongue motion leading to treatment outcomes.

**Figure S2:** Ultrasonography imaging setup.

**Figure S3:** Therapeutic voltage and tongue base movement. The therapeutic voltage is displayed against anterior tongue base movement of the implanted side in the axial plane (A), anterior tongue base movement in the sagittal plane (B), and bilateral tongue base movement (C). A locally estimated scatterplot smoothing (LOESS) with a 95% confidence interval (gray shading) illustrates the trend.

**Figure S4:** Therapeutic voltage and buckling and trough sign. The therapeutic voltage is displayed against buckling (A), and trough sign (B). A diamond shape indicates the mean.

**Table S1:** Sensitivity analysis of non‐supine (A) and supine (B) apnea‐hypopnea index reduction and tongue base motion.

**Table S2:** Sensitivity analysis of responder only and tongue base motion.

**Video S1:** Supporting Information video: Video illustrating the various tongue movement patterns.

**Data S1:** Supporting Information methods: worksheet for tongue movement assessment.

## Data Availability

The data that support the findings of this study are available on request from the corresponding author. The data are not publicly available due to privacy or ethical restrictions.
