# Supplementary figures and images for: Ultrasonographic Tongue Base Motion Does Not Correlate With Hypoglossal Nerve Stimulation Outcomes
Source: Laryngoscope Investig Otolaryngol. 2026 Mar 10;11(2):e70376. doi: 10.1002/lio2.70376 (PMC12976454; doi:10.1002/lio2.70376)

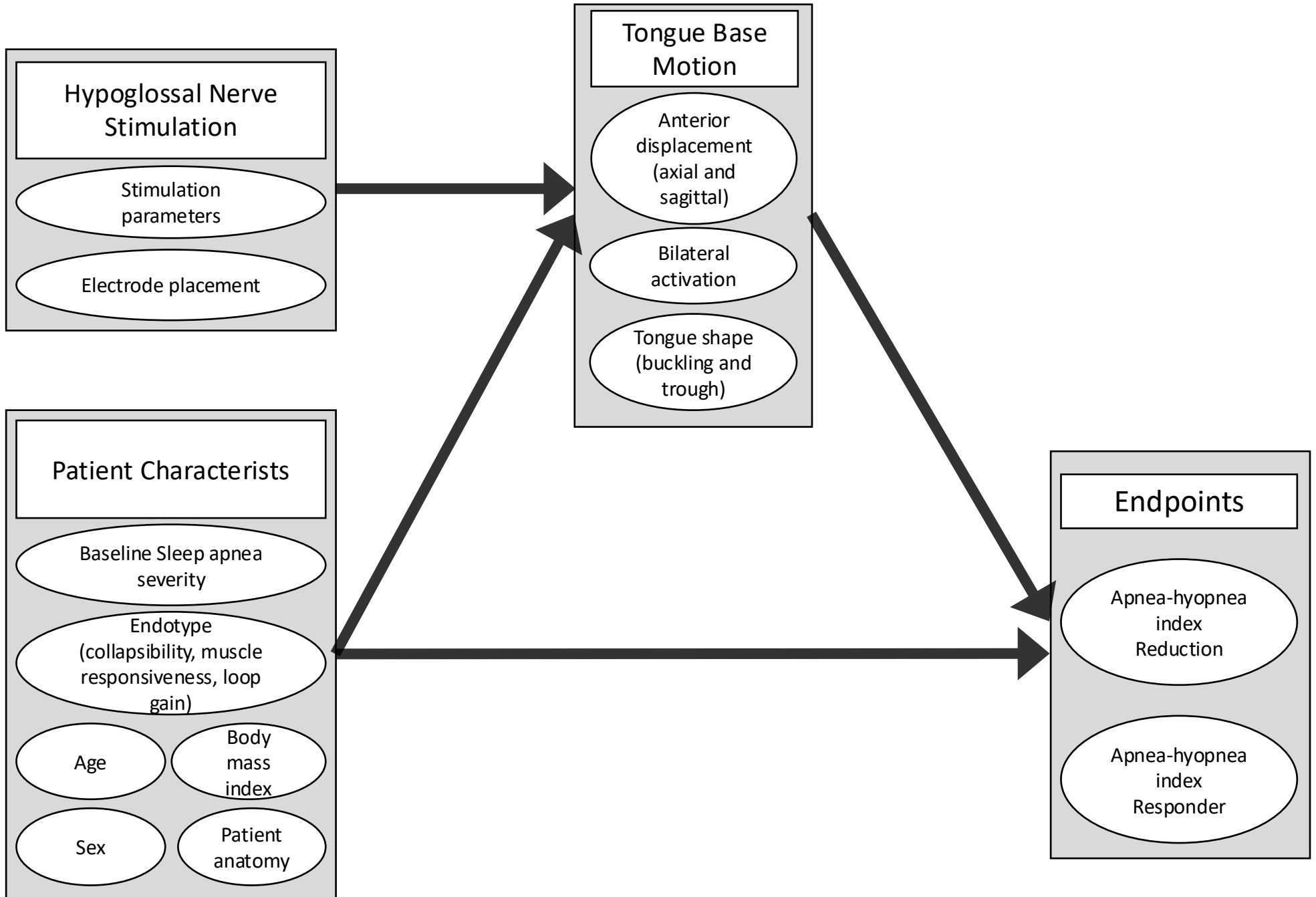

Supplement: Supplementary file 1 — Figure S1: Directed acyclic graph illustrating the assumed causal relationships between baseline patient characteristics, hypoglossal nerve stimulation, and tongue motion leading to treatment outcomes. [file LIO2-11-e70376-s004.pdf]

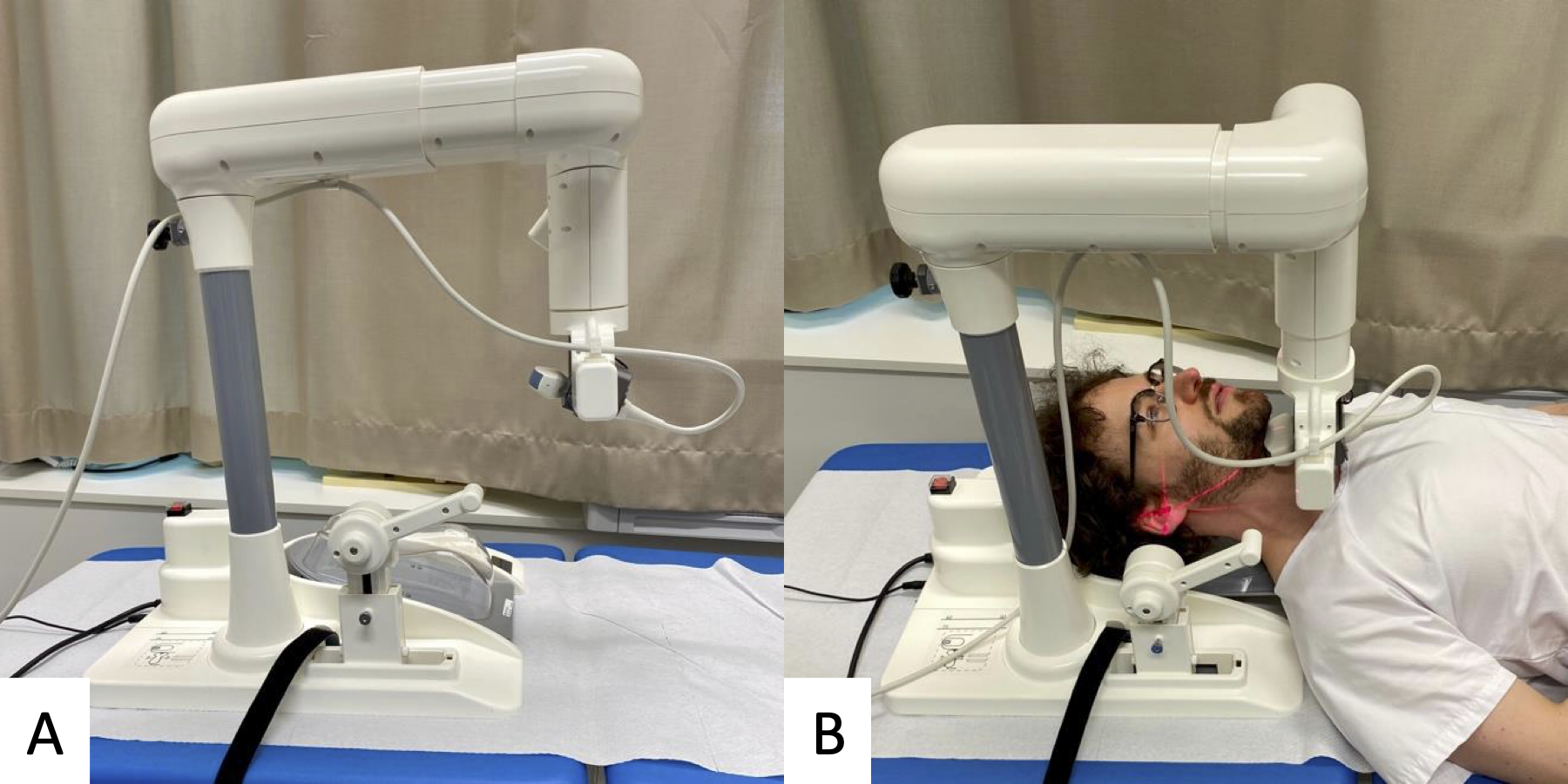

Supplement: Supplementary file 2 — Figure S2: Ultrasonography imaging setup. [file LIO2-11-e70376-s005.tiff]

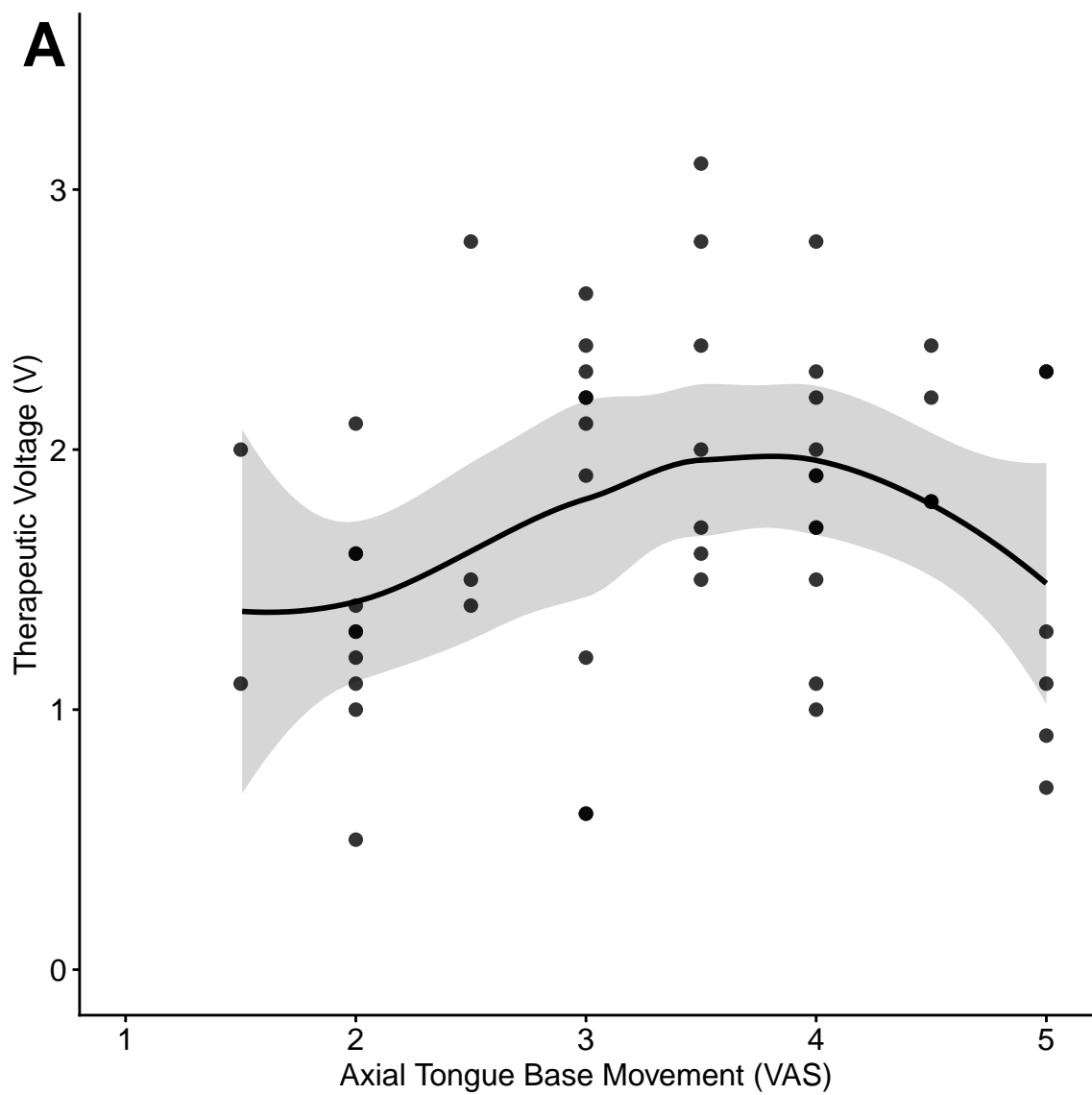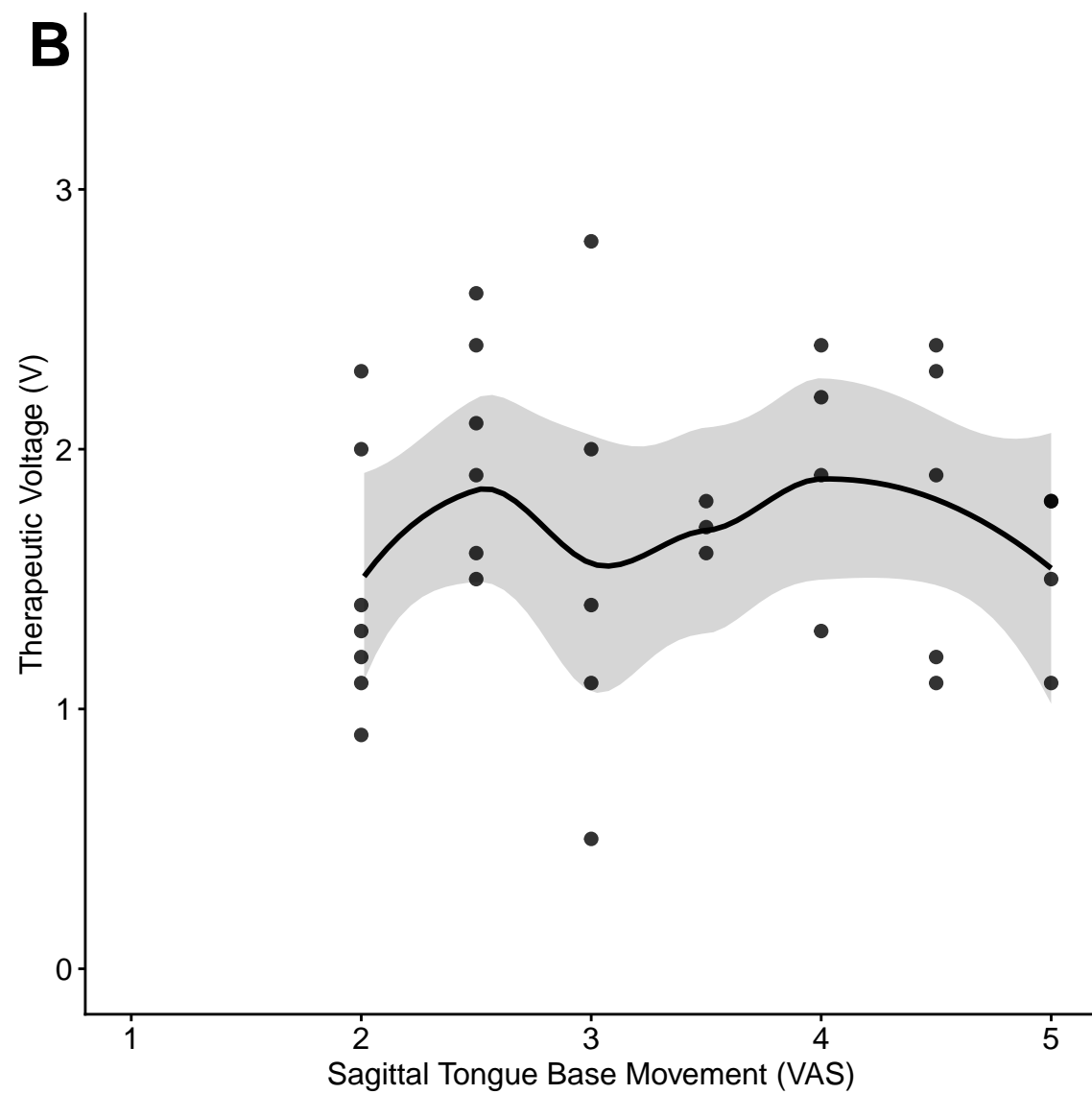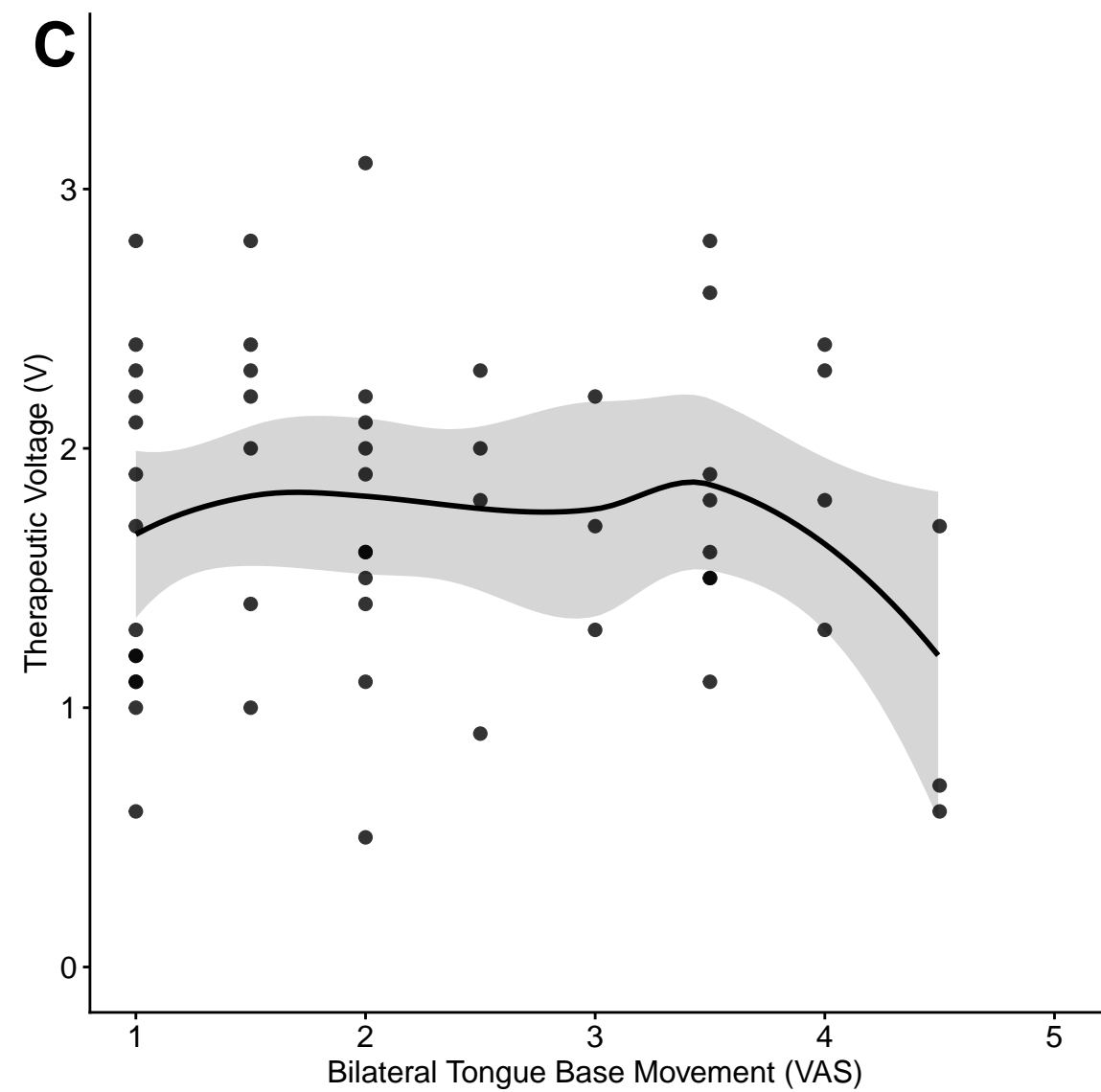

Supplement: Supplementary file 3 — Figure S3: Therapeutic voltage and tongue base movement. The therapeutic voltage is displayed against anterior tongue base movement of the implanted side in the axial plane (A), anterior tongue base movement in the sagittal plane (B), and bilateral tongue base movement (C). A locally estimated scatterplot smoothing (LOESS) with a 95% confidence interval (gray shading) illustrates the trend. [file LIO2-11-e70376-s003.pdf]

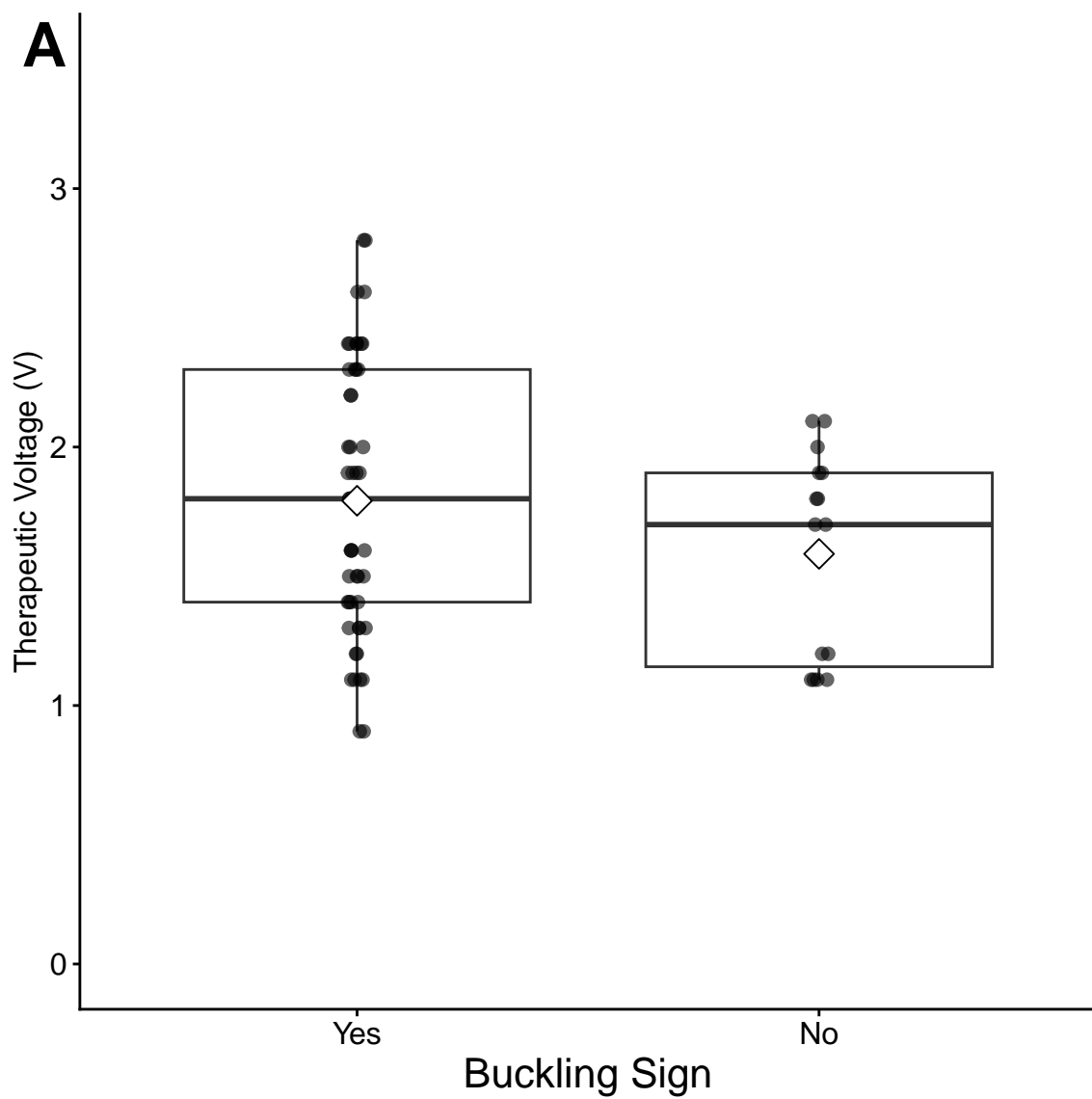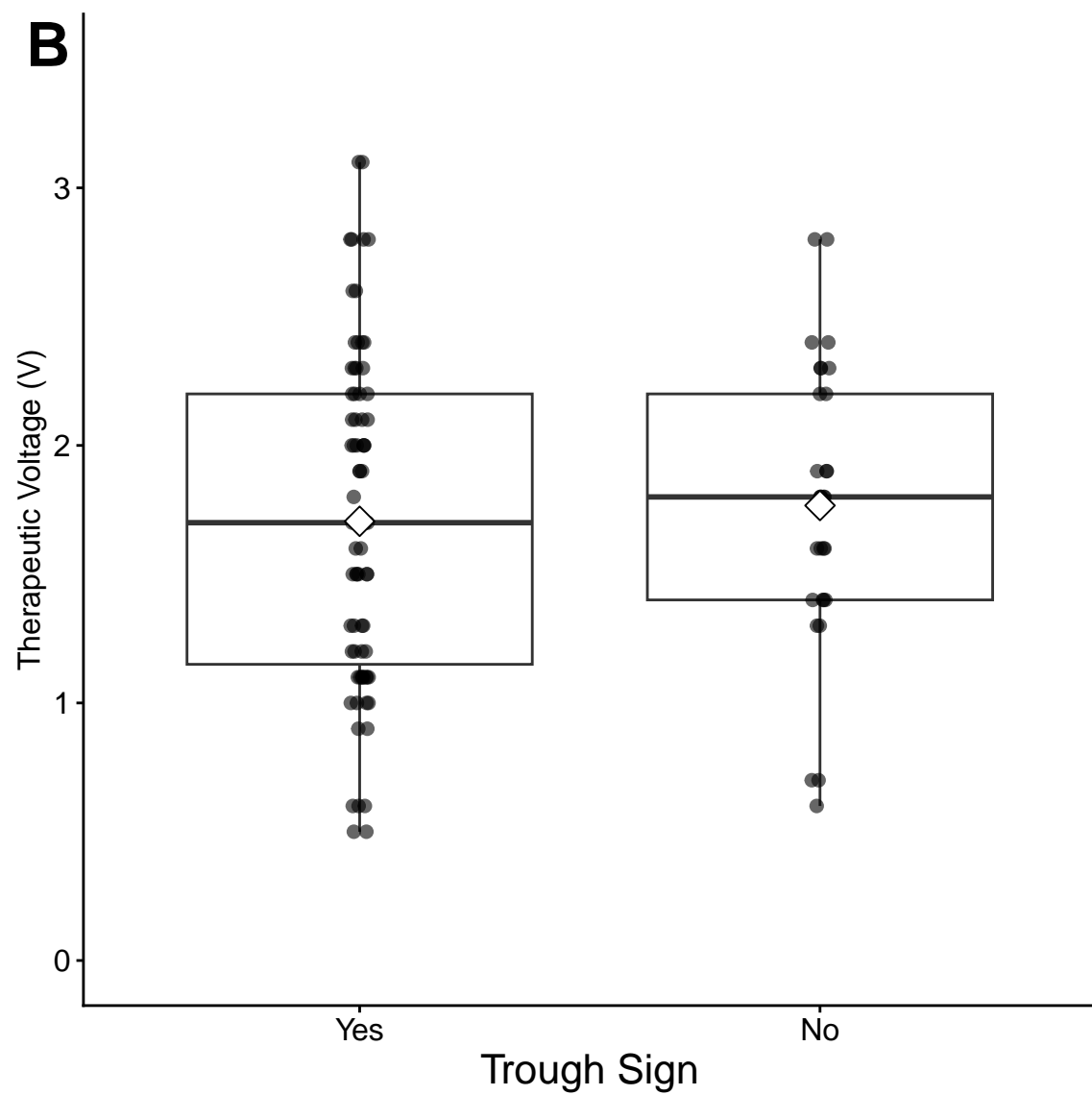

Supplement: Supplementary file 4 — Figure S4: Therapeutic voltage and buckling and trough sign. The therapeutic voltage is displayed against buckling (A), and trough sign (B). A diamond shape indicates the mean. [file LIO2-11-e70376-s001.pdf]
