# Supplementary material for: Ultrasonographic Tongue Base Motion Does Not Correlate With Hypoglossal Nerve Stimulation Outcomes
Source: Laryngoscope Investig Otolaryngol. 2026 Mar 10;11(2):e70376. doi: 10.1002/lio2.70376 (PMC12976454; doi:10.1002/lio2.70376)
Supplement: Supplementary file 5 — Table S1: Sensitivity analysis of non‐supine (A) and supine (B) apnea‐hypopnea index reduction and tongue base motion. [file LIO2-11-e70376-s008.docx]

| **Predictor** | **Effect (95% confidence interval)** | **P value** | **P value (adjusted)** |
| --- | --- | --- | --- |
| Sagital Tongue Base Movement (VAS) | 0.22 [-0.22, 0.59] | 0.321 | 0.782 |
| Axial Tongue Base Movement (VAS) | 0.08 [-0.24, 0.39] | 0.626 | 0.782 |
| Bilateral Tongue Base Movement (VAS) | 0.01 [-0.31, 0.33] | 0.952 | 0.952 |
| Buckling Sign | 6.59 [-20.99, 41.41] | 0.622 | 0.782 |
| Trough Sign | 4.99 [-10.89, 26.13] | 0.557 | 0.782 |

**Supporting Information Table S1A:** Sensitivity Analysis of non-supine apnea-hypopnea index reduction and tongue base motion.

Effect sizes are presented as Spearman’s rank correlation coefficients or Hodges–Lehmann median differences, each with corresponding 95% confidence intervals. P values were adjusted for multiple testing using the Benjamini–Hochberg false discovery rate procedure.

**Supporting Information Table S1B:** Sensitivity Analysis of supine apnea-hypopnea index reduction and tongue base motion.

| **Predictor** | **Effect (95% confidence interval)** | **P value** | **P value (adjusted)** |
| --- | --- | --- | --- |
| Sagital Tongue Base Movement (VAS) | -0.17 [-0.54, 0.26] | 0.438 | 0.634 |
| Axial Tongue Base Movement (VAS) | 0.13 [-0.18, 0.41] | 0.407 | 0.634 |
| Bilateral Tongue Base Movement (VAS) | 0.03 [-0.27, 0.33] | 0.842 | 0.842 |
| Buckling Sign | 16.74 [-22.58, 41.19] | 0.308 | 0.634 |
| Trough Sign | 3.80 [-12.85, 19.60] | 0.507 | 0.634 |

Effect sizes are presented as Spearman’s rank correlation coefficients or Hodges–Lehmann median differences, each with corresponding 95% confidence intervals. P values were adjusted for multiple testing using the Benjamini–Hochberg false discovery rate procedure.
