# Supplementary material for: Ultrasonographic Tongue Base Motion Does Not Correlate With Hypoglossal Nerve Stimulation Outcomes
Source: Laryngoscope Investig Otolaryngol. 2026 Mar 10;11(2):e70376. doi: 10.1002/lio2.70376 (PMC12976454; doi:10.1002/lio2.70376)
Supplement: Supplementary file 6 — Table S2: Sensitivity analysis of responder only and tongue base motion. [file LIO2-11-e70376-s007.docx]

**Supporting Information Table S2:** Sensitivity Analysis of apnea-hypopnea index reduction and tongue base motion including only responders to treatment.

| **Predictor** | **Effect (95% confidence interval)** | **p value** | **p value (adjusted)** |
| --- | --- | --- | --- |
| Sagital Tongue Base Movement (VAS) | -0.22 [-0.64, 0.29] | 0.391 | 0.944 |
| Axial Tongue Base Movement (VAS) | -0.09 [-0.45, 0.30] | 0.663 | 0.944 |
| Bilateral Tongue Base Movement (VAS) | -0.06 [-0.43, 0.32] | 0.755 | 0.944 |
| Buckling Sign | -0.32 [-16.10, 32.40] | 1.000 | 1.000 |
| Trough Sign | 2.47 [-5.10, 12.30] | 0.421 | 0.944 |

Effect sizes are presented as Spearman’s rank correlation coefficients or Hodges–Lehmann median differences, each with corresponding 95% confidence intervals. P values were adjusted for multiple testing using the Benjamini–Hochberg false discovery rate procedure.
