# Supplementary material for: Ultrasonographic Tongue Base Motion Does Not Correlate With Hypoglossal Nerve Stimulation Outcomes
Source: Laryngoscope Investig Otolaryngol. 2026 Mar 10;11(2):e70376. doi: 10.1002/lio2.70376 (PMC12976454; doi:10.1002/lio2.70376)
Supplement: Supplementary file 8 — Data S1: Supporting Information methods: worksheet for tongue movement assessment. [file LIO2-11-e70376-s006.docx]

**Supplementary Information Methods:**

**Ultrasonographic Tongue Base Motion Does Not Correlate with Hypoglossal Nerve Stimulation Outcomes**

| 1  None | 2  Stiffening / minimal movement | 3  Some movement | 4  Good movement | 5  Excellent movement |
| --- | --- | --- | --- | --- |
| 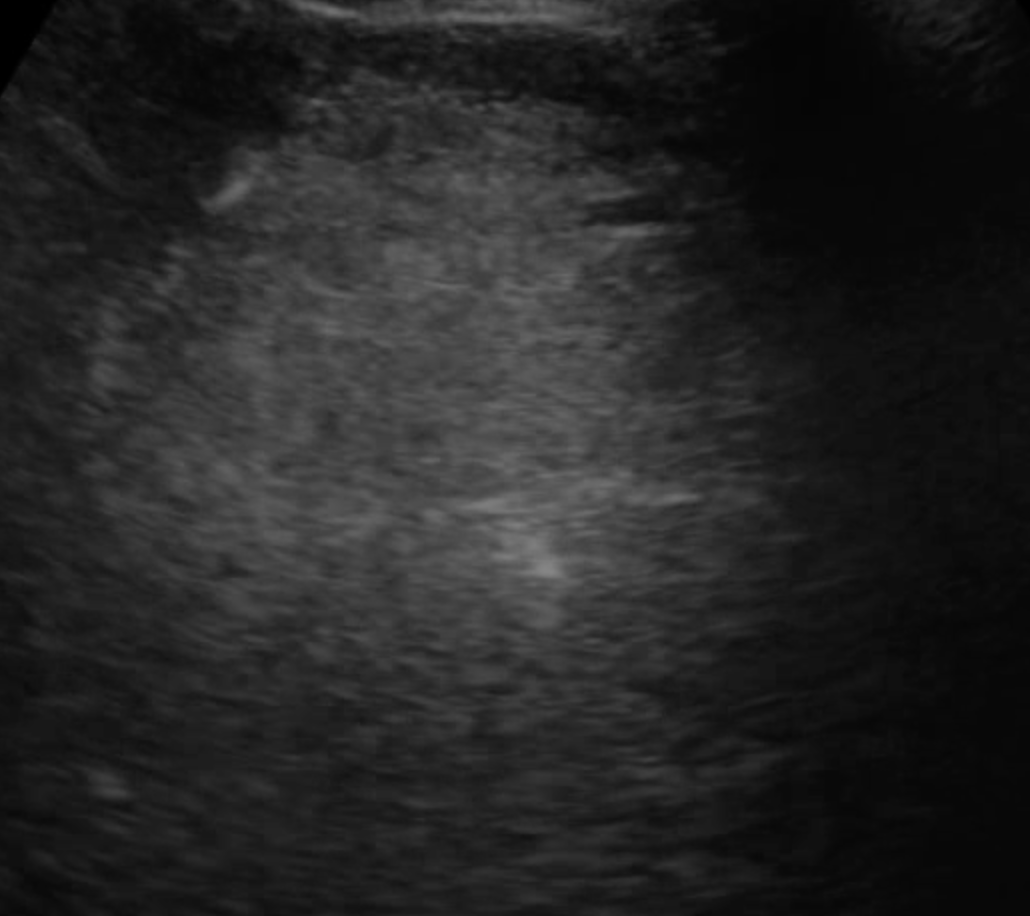 |  | 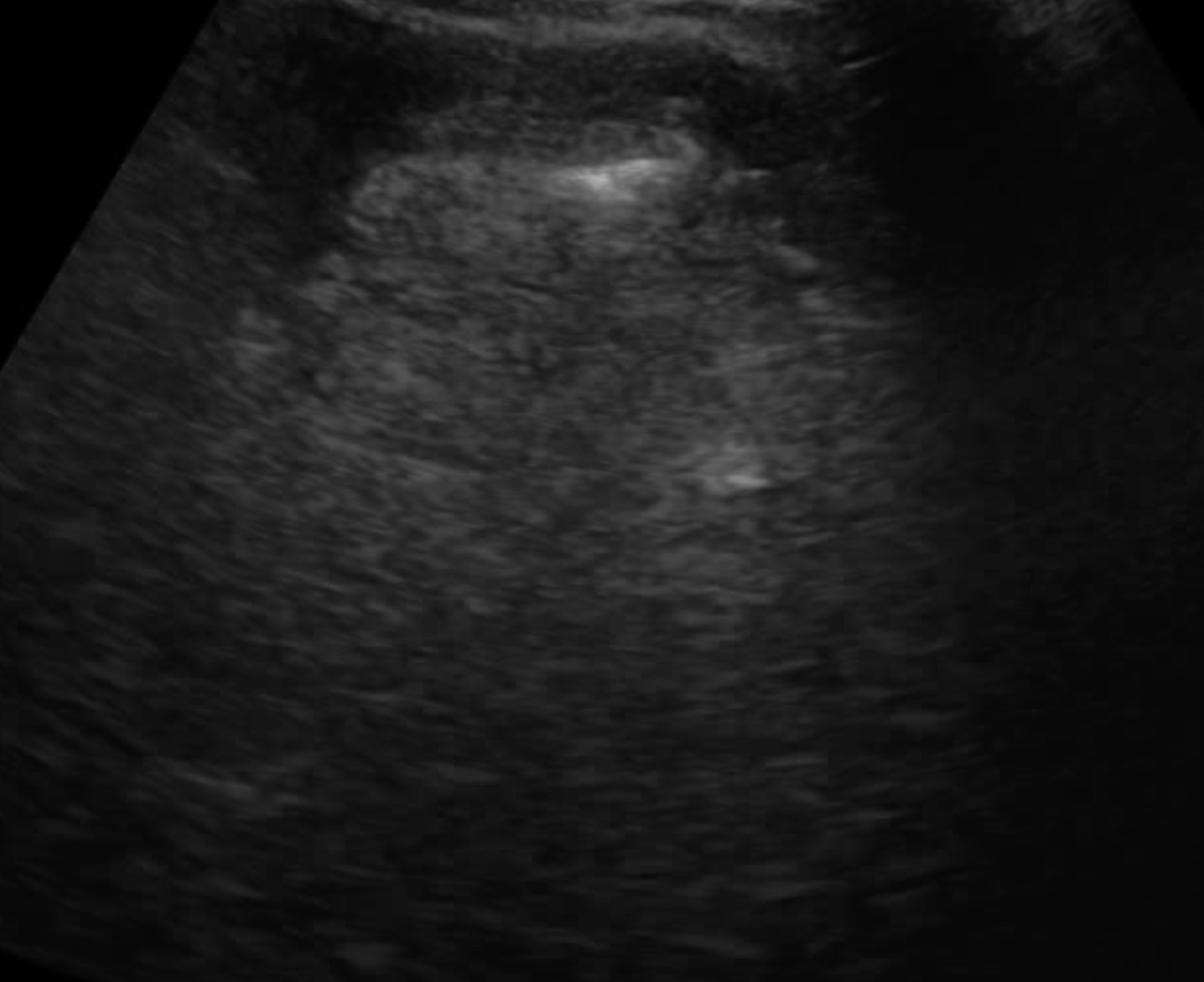 |  | 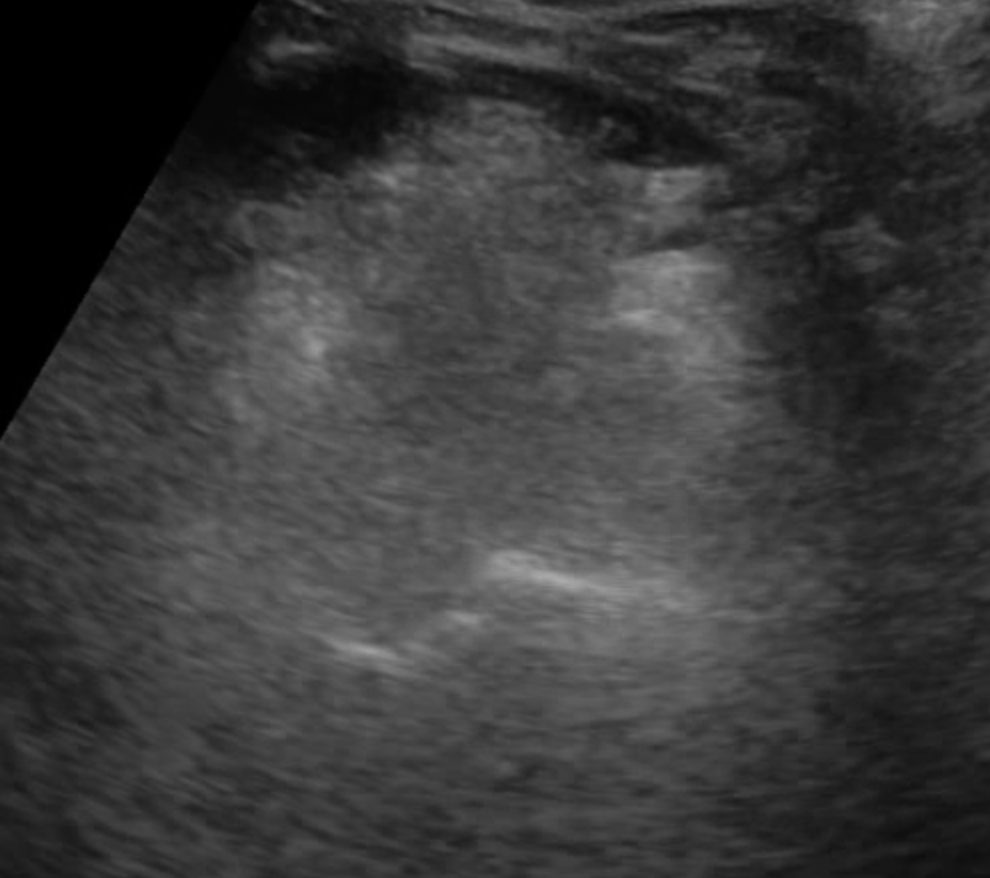 |

**Tongue Base Movement of Implanted Side in the Axial Plane**

**Bilateral Tongue Base Movement**

| 1  Isolated unilateral | 2 | 3  Some asymmetry | 4 | 5  Bilateral movement |
| --- | --- | --- | --- | --- |
| 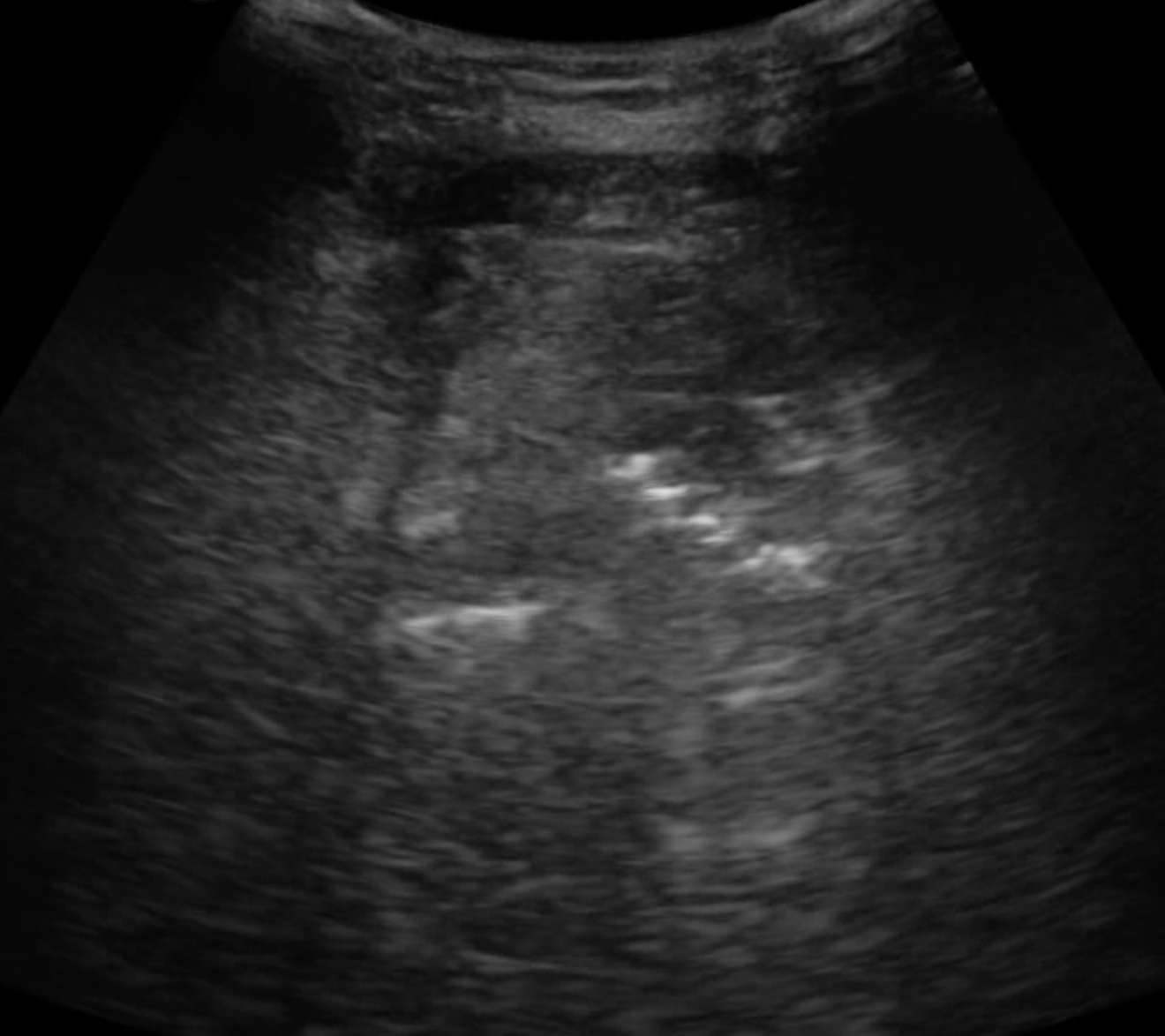 |  | 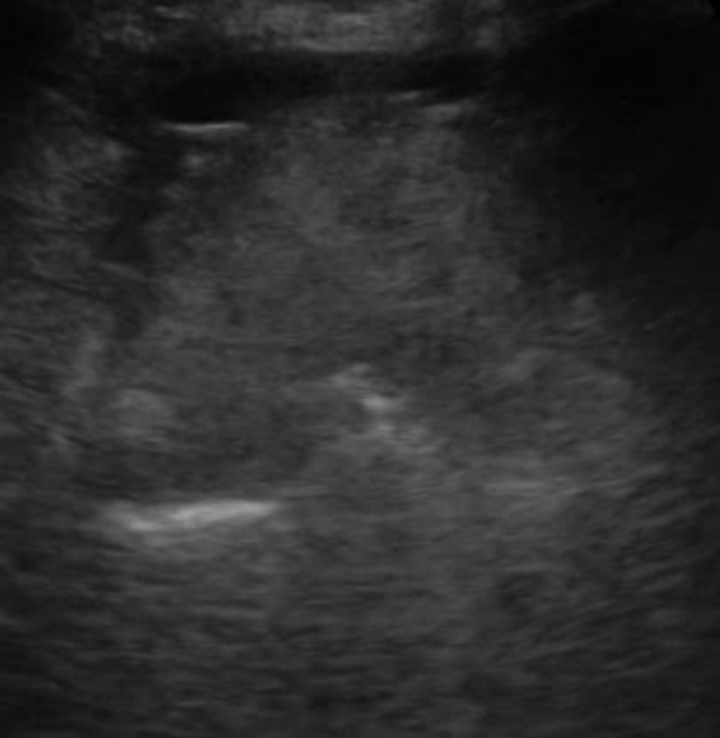 |  | 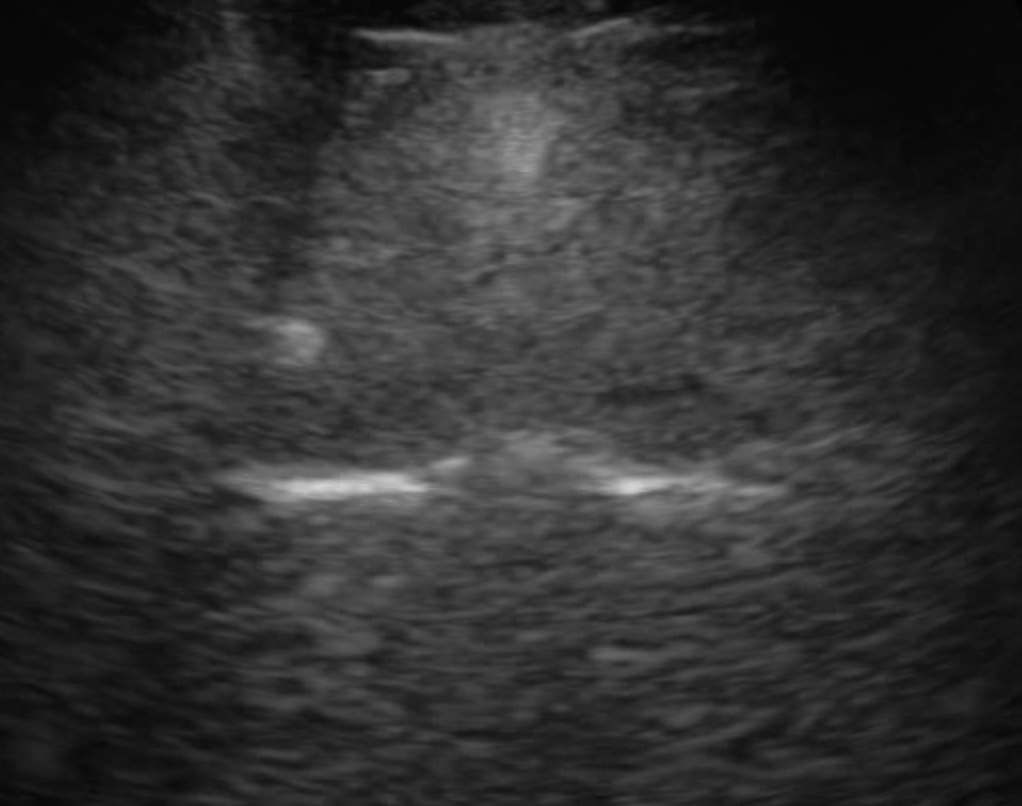 |

| 1  None | 2  Stiffening / minimal movement | 3  Some movement | 4  Good protrusion | 5  Excellent protrusion |
| --- | --- | --- | --- | --- |
| **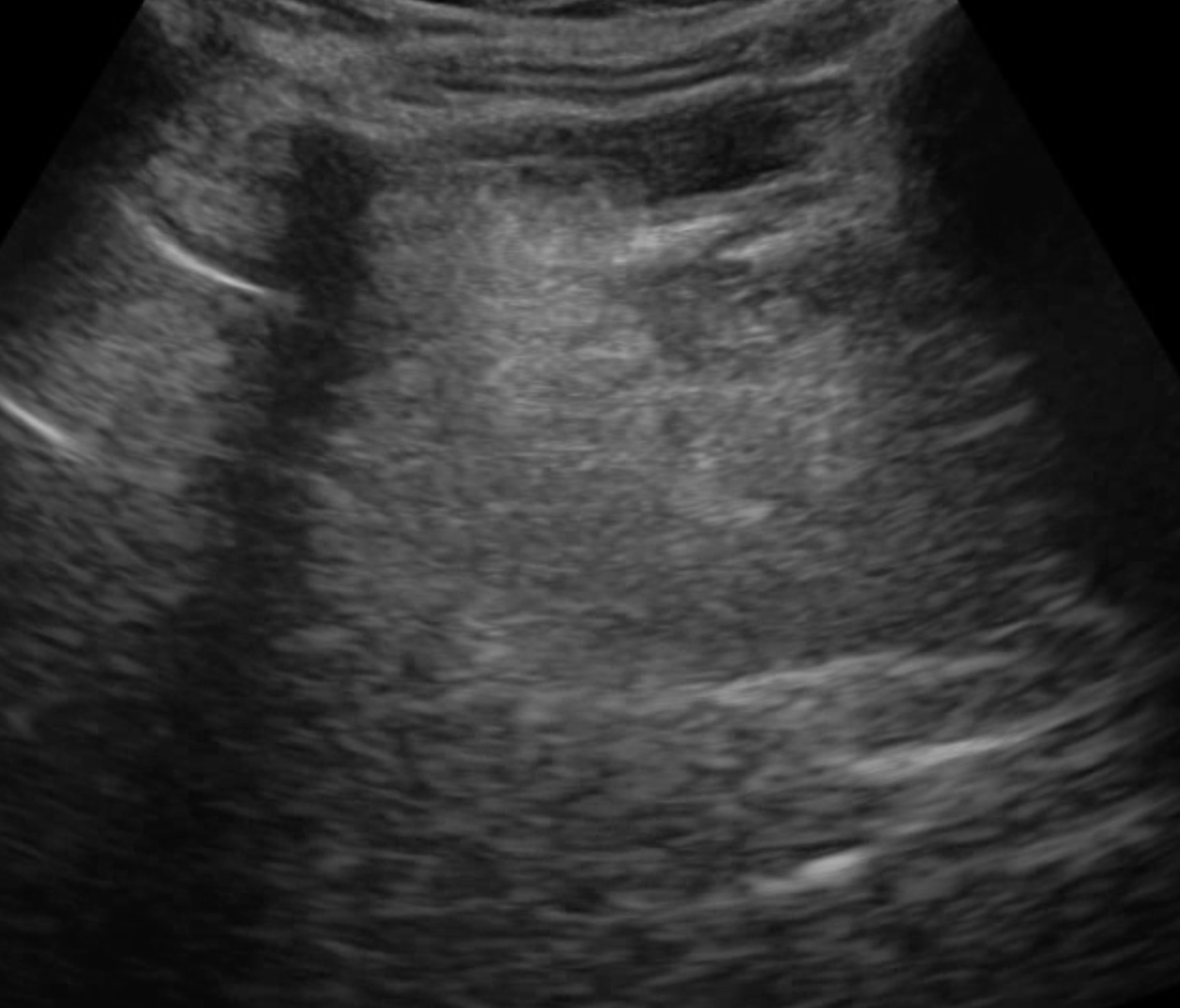** |  | **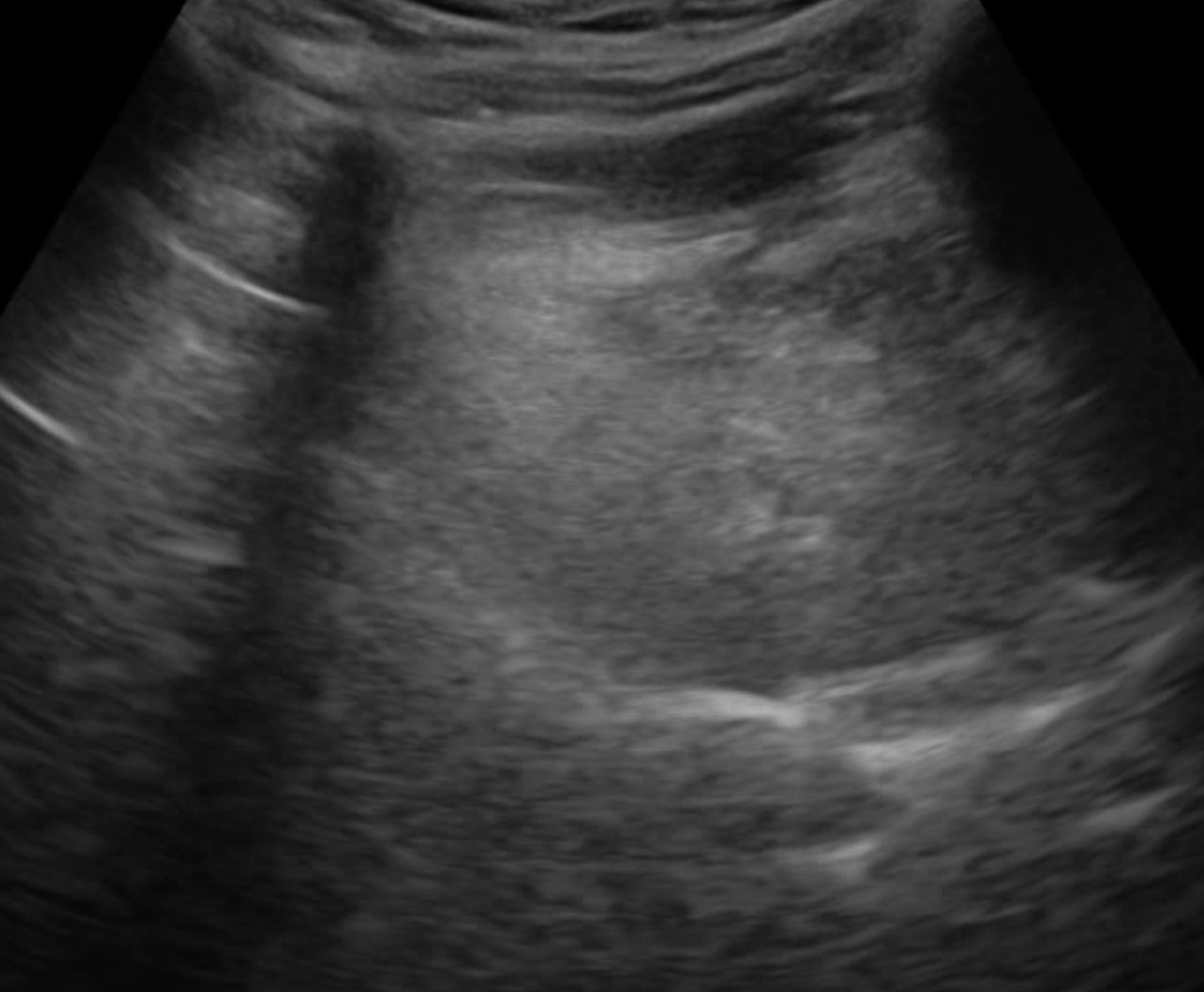** |  | **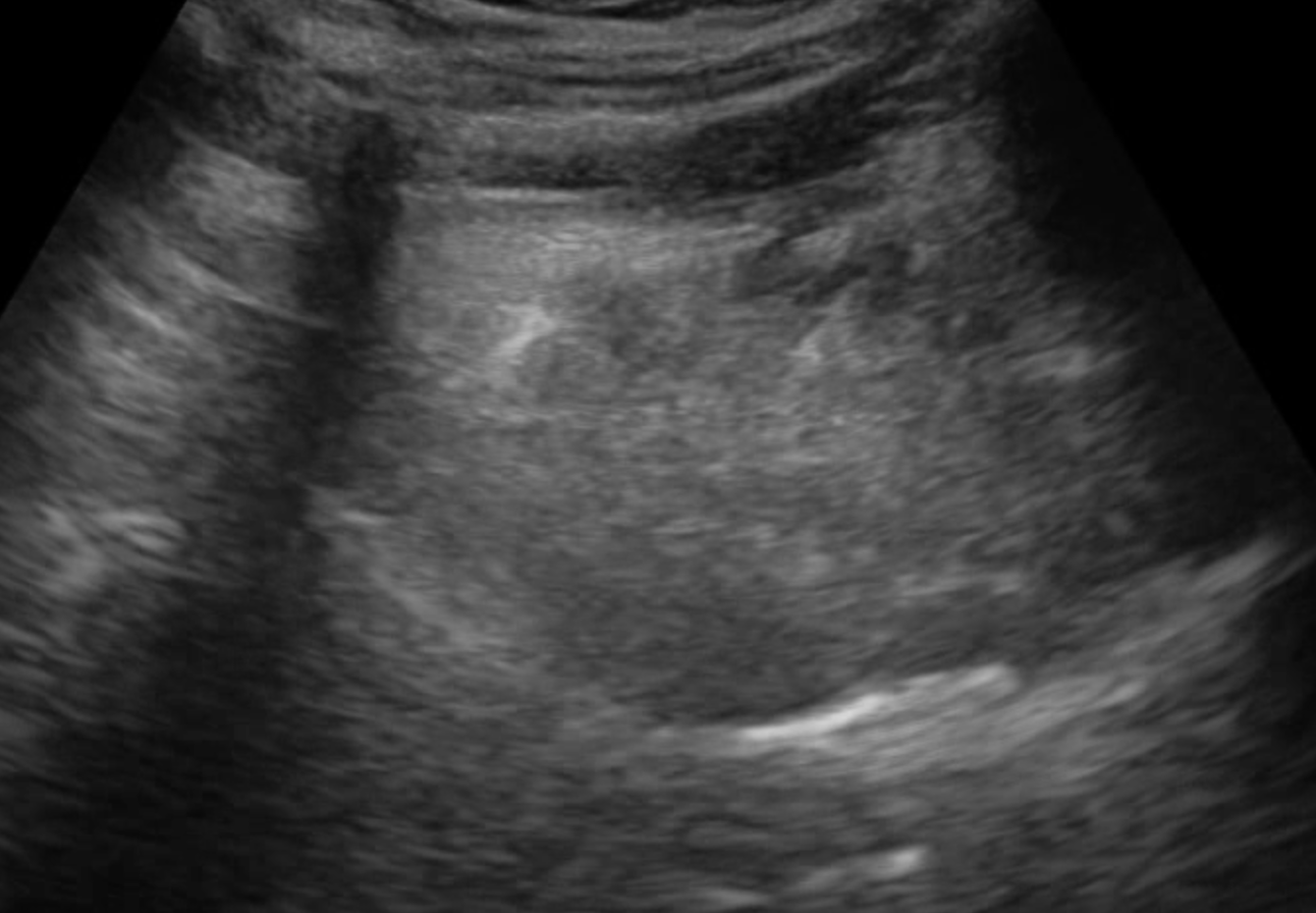** |

**Tongue Base Protrusion in the Sagittal Plane**

**Trough Sign**
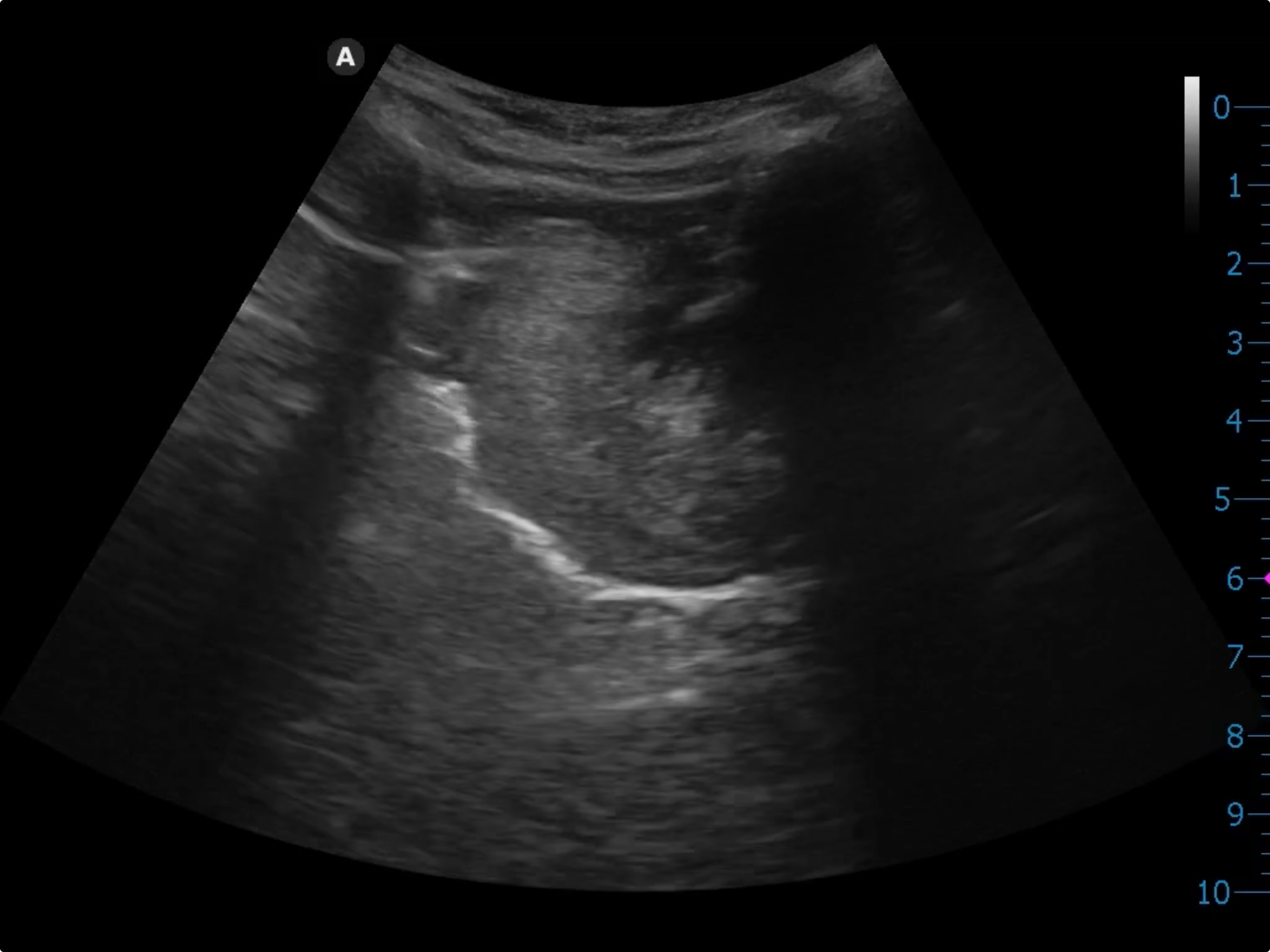


Trough sign was defined as a V-shape of the tongue base to airway interface during stimulation in the axial plane.

**Buckling**


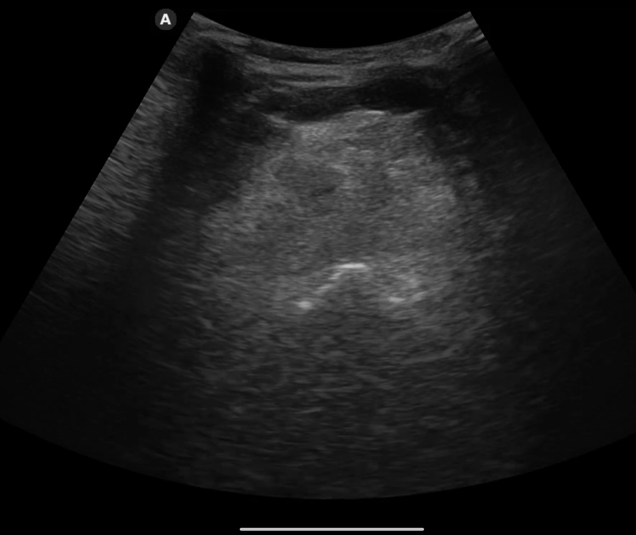


Buckling was defined as an increase in tongue height rather than protrusion during stimulation in the sagittal plane.
